# Strategies in primary healthcare to implement early identification of risky alcohol consumption: why do they work or not? A qualitative evaluation of the ODHIN study

**DOI:** 10.1186/s12875-016-0461-8

**Published:** 2016-06-07

**Authors:** M. Keurhorst, M. Heinen, J. Colom, C. Linderoth, U. Müssener, K. Okulicz-Kozaryn, J. Palacio-Vieira, L. Segura, F. Silfversparre, L. Słodownik, E. Sorribes, M. Laurant, M. Wensing

**Affiliations:** Radboud university medical center, Radboud Institute for Health Sciences, IQ healthcare, Nijmegen, The Netherlands; Saxion University of Applied Sciences, Centre for Nursing Research, Deventer/Enschede, The Netherlands; Program on Substance Abuse, Public Health Agency, Government of Catalonia, Barcelona, Spain; Department of Medicine and Health Sciences, Linköping University, Linköping, Sweden; State Agency for Prevention of Alcohol-Related Problems, Warsaw, Poland; The Section for Epidemiology and Social Medicine, University of Gothenburg, Gothenburg, Sweden; HAN University of Applied Sciences, Faculty of Health and Social Studies, Nijmegen, The Netherlands

**Keywords:** Screening and brief intervention, Alcohol prevention, Primary healthcare, Implementation, Qualitative evaluation

## Abstract

**Background:**

Screening and brief interventions (SBI) in primary healthcare are cost-effective in risky drinkers, yet they are not offered to all eligible patients. This qualitative study aimed to provide more insight into the factors and mechanisms of *why, how, for whom* and *under what circumstances* implementation strategies work or do not work in increasing SBI.

**Methods:**

Semi-structured interviews were conducted between February and July 2014 with 40 GPs and 28 nurses in Catalonia, the Netherlands, Poland, and Sweden. Participants were purposefully selected from the European Optimising Delivery of Healthcare Interventions (ODHIN) trial. This randomised controlled trial evaluated the influence of training and support, financial reimbursement and an internet-based method of delivering advice on SBI. Amongst them were 38 providers with a high screening performance and 30 with a low screening performance from different allocation groups. Realist evaluation was combined with the Tailored Implementation for Chronic Diseases framework for identification of implementation determinants to guide the interviews and analysis. Transcripts were analysed thematically with the diagram affinity method.

**Results:**

Training and support motivated SBI by improved knowledge, skills and prioritisation. Continuous provision, sufficient time to learn intervention techniques and to tailor to individual experienced barriers, seemed important T&S conditions. Catalan and Polish professionals perceived financial reimbursement to be an additional stimulating factor as well, as effects on SBI were smoothened by personnel levels and salary levels. Structural payment for preventive services rather than a temporary project based payment, might have increased the effects of financial reimbursement. Implementing e-BI seem to require more guidance than was delivered in ODHIN. Despite the allocation, important preconditions for SBI routine seemed frequent exposure of this topic in media and guidelines, SBI facilitating information systems, and having SBI in protocol-led care. Hence, the second order analysis revealed that the applied implementation strategies have high potential on the micro professional level and meso-organisational level, however due to influences from the macro- level such as societal and political culture the effects risks to get nullified.

**Conclusions:**

Essential determinants perceived for the implementation of SBI routines were identified, in particular for training and support and financial reimbursement. However, focusing only on the primary healthcare setting seems insufficient and a more integrated SBI culture, together with meso- and macro-focused implementation process is requested.

**Trial registration:**

ClinicalTrials.gov. Trial identifier: NCT01501552.

## Background

Alcohol consumption is a substantial contributor to the global burden of disease. It is a leading factor for more than 200 diseases, injuries and other health conditions with ICD-10 codes [1]. The highest levels of alcohol consumption can be found in the European Union with approximately eleven litres alcohol per capita per year [1]. Evidence shows that 20–30 % of patients who present in primary healthcare are risky drinkers [2]. Several meta-analyses have shown that simple screening consisting of a few standardised questions, followed by a brief counselling intervention (consisting of simple advice or psychological counselling) significantly reduces alcohol consumption in primary healthcare populations [3–6]. However, there is a large gap between patients’ needs and the actual provision of advice. In current European primary healthcare settings [7, 8] less than 10 % of the population at risk are identified, and less than 5 % of those who could benefit are offered screening and brief advice. Furthermore, alcohol is the least discussed lifestyle theme compared to smoking, physical activity and dietary habits in Dutch primary healthcare [9].

Barriers for screening and brief intervention (SBI) delivery by primary healthcare professionals have been identified in previous research and primarily comprised lack of knowledge in health providers; lack of adequate resources and support; and, time constrains in terms of perceived workload for SBI [10–12].

An increasing number of studies are being conducted in primary healthcare to stimulate the uptake of SBI for risky alcohol consumption (i.e. implementation strategies) [2, 13, 14], albeit with very limited success. The effectiveness of these so-called implementation strategies are summarised in several reviews [15–17]. In short, these reviews found that effectiveness of implementation programmes on SBI delivery increases when they are multi-component [15], contain higher intensity effort [16], and focus on GP’s and mid-level professionals simultaneously [17]. These enablers of improvements are known as determinants of practice. The detailed process of these enablers in reaching actual uptake of SBI for risky alcohol consumption, are described in mechanisms of change [18]. More insight into determinants and actual mechanisms of change would help to tailor implementation programmes to key issues [18]. There are several qualitative studies conducted on barriers and facilitators for SBI delivery (e.g. [19–21]), although these give limited empirical insight into determinants of practice and mechanisms of change while implementing SBI in daily practice. This qualitative study was conducted after a controlled randomised trial to provide more insight into the factors and mechanisms of SBI implementation for risky alcohol consumption in primary healthcare. Linking theoretical knowledge from the implementation science database to practice-led experiences, views and attitudes from primary healthcare providers would add important knowledge on the current implementation gap. Therefore, the purpose of this qualitative study is to explore according to professionals’ views on *why, how, for whom* and *under what circumstances* implementation strategies worked or did not work in increasing SBI.

## Methods

### Study design

We conducted a qualitative study with realist evaluation as methodological orientation after the Optimising Delivery of Healthcare Interventions (ODHIN) randomised controlled trial [22]. The ODHIN study attempted to overcome barriers for primary healthcare professional change by testing three different implementation strategies in a cluster randomised factorial trial in five European countries that represent the European alcohol levels (England, Catalonia, Sweden, Poland and the Netherlands). These countries differed in their organisation of primary care and their drinking patterns so the precise content of the implementation strategies were fine-tuned to country contexts. With regard to the lack of knowledge in healthcare professionals, we applied a training and support (T&S) implementation programme. In this programme the professionals’ role security and therapeutic commitment were taken into account in order to address issues during training and support. The programme consisted of two initial 1–2 h face-to-face educational trainings, and one (10–30 min) telephone support call. With regard to lack of resources and support, we applied country-dependent financial reimbursement (FR) schemes. FR concerned payment for screening and advice activities, with rates based on existing country-specific financial reimbursement for clinical preventive activities. Finally, perceived workload was addressed by an internet-based method of delivering advice (e-BI) instead of face-to-face brief interventions to save professionals’ time [22]. In the trial, these strategies were tested in every possible combination and resulted consequently in eight allocation groups. The perspective of the Realist Evaluation [23, 24] is an approach that originates from educational research. The core of this approach were the ‘how’ and ‘why’ questions [23], which fitted our research question of evaluating the implementation strategies applied in the ODHIN study. From this perspective, we sought to establish what worked, for whom, in what circumstances, in what respect, to what extent, and why. Our focus thereby was on the processes by which the ODHIN trial achieved its outcomes. Its starting point was that it was not only the implementation strategy that changed professional behaviours or processes, but also the participants’ reaction to the opportunities provided by the programme that triggered the change, in combination with reinforcing or hindering factors outside the programme [23].

The consolidated criteria for reporting qualitative research (COREQ-32) [25] were used to design and report the current study.

Ethics approval for the study was obtained from the relevant approval bodies within each country: In Catalonia, the Clinical Research Ethics Committee of the Jordi Gol I Gurina Primary Health Care Research Institute and from the Clinical Research Ethics Committee of Hospital Clínic de Barcelona; in Poland, Resolution No. KB- 0012/105/11 adopted by the Commission of Bioethics of the Pomeranian Medical University in Szczecin; and, in Sweden by the: Regional Ethical Review Board in Göteborg, reference number: 658/12, with approval granted for both sites in Göteborg and Linköping. In the Netherlands, the Committee on Research inv. Human Subjects (CMO) ethical board declared that no ethical approval was required in the Netherlands, reference number: 2012/281. In all four countries, all participating healthcare providers signed a written informed consent and the interviews did not place burdens on the participants.

### Framework analysis

The ‘Tailored Implementation for Chronic Diseases’ framework (TICD) [18] was used in applying framework analysis. The TICD framework was primarily developed to implement changes in prevention and chronic disease management in primary healthcare, and is through a systematic review and consensus process based on an integrative analysis of 14 previously published frameworks, theories and models. The framework includes seven domains of implementation determinants: 1) guideline factors; 2) individual health professional factors; 3) patient factors; 4) professional interactions; 5) incentives and resources; 6) capacity for organisational change; 7) social, political and legal factors. The framework is designed to understand change of professional behaviour and organisation of practice [18] and was applied as an organising principle. Consequently, the framework was relevant in this more structured approach to qualitative data analysis, in order to build on previous body of research in barriers for implementation of evidence-based practice. Besides, it provides room to add concepts, other than already existing in the framework. This flexibility was relevant in facilitating the ‘open’ nature of the topic guide, which is provided below.

### Participants and setting

Of the five trial countries, only England was not able to participate due to lack of funding. From the 96 participating Catalan, Swedish, Polish and Dutch primary healthcare units (PHCU), each country research team invited ODHIN participating professionals to participate to the qualitative study. The recruitment of individuals was based on purposive sampling throughout a range of maximum variation, to receive insight into why, how, for whom and under what circumstances the implementation strategies work. The sampling was based on three features:

1. occupation: GP or nurse, although in Poland only GPs were invited as no nurses participated in the trial [22]
2. screening performance after receiving implementation strategies: professionals with upper quartile versus lowest quartile of country screening rates. The screening rate was calculated as the number of completed screens divided by the total number of consultations of all patients eligible for screening.
3. implementation strategy: T&S versus no T&S. The T&S group includes professionals from 4 allocation groups: T&S alone, T&S + FR, T&S + e-BI and T&S + FR + e-BI. The non-T&S group includes professionals from the other four allocation groups: FR alone, e-BI alone, FR + e-BI, and no strategy. This sampling criterion ensured that professionals who received these different types of strategies were equally included in our study sample.

Professionals were invited by mail and by telephone. In case of non-response after email, we invited professionals directly by phone and planned the interviews.

### Data collection

Interviews were performed between February and July 2014 by ODHIN trial researchers and focused on all three implementation strategies. Furthermore, field notes were made during and after the interviews. Researchers in different countries varied somewhat in posing their questions about the three strategies. Sweden and the Netherlands pro-actively asked professionals about experiences with all three implementation strategies. Catalonia covered all three but focused on T&S, whereas Poland mainly focused on the project generally and asked for further explanation when any of the strategies was raised by the professionals themselves.

We conducted semi-structured individual interviews by telephone using interview guides and topic lists developed for this study. No other people were present at the time of the interviews, these were conducted in private rooms. Topic lists were piloted and revised according to the results of the first interviews in each of the countries. Both the realist evaluation perspective and TICD framework served as a guide in developing the topic list (the interview guide is available on request):

- Why?
  - o Engagement: reasons for subscribing to the ODHIN trial
- How and for whom?
  - o Description of the SBI implementation process: description of SBI proceedings and expectations
- Under what circumstances?
  - o Barriers and facilitators to following the guidelines on risky alcohol consumption
  - o Facilitators or barriers to implementing SBI, related to the allocation groups
  - o Opinions and suggestions for organisational and political barriers and facilitators
  - o Other thoughts and suggestions to speed up the implementation process

All interviews were audio taped, transcribed verbatim in each country’s native language and anonymised.

### Data analysis

The analysis consisted of four phases. First, each country coded independently - at least two researchers from each country independently coded fragments of the transcripts inductively and with constant discussion on interpretations, into English codes to facilitate building an international code book [26]. In this way, country researchers discussed on national and on international level their interpretation of the interviews, exchanged their views and came to an agreement for the appropriate code for the international code book. This final code book covered national as well as international interpretations, which allowed codes applied in single countries. Data collection and data analyses were alternated. Creditability was addressed by checking findings from analysis by further interviews. Furthermore, the research team included general practitioners and nurses as well. Each country used software and methods that they were familiar with, i.e. Atlas.ti version 7.1.5 (ATLAS.ti Scientific Software Development Company, GmbH, Berlin, Germany), Nvivo 10 or Microsoft Word to facilitate the coding process. Codes were structured by the seven broad TICD framework domains [18], followed by an open coding procedure, resulting in a largely inductive content analysis. When codes could not be structured by one of the seven TICD domains, they were organised in an eighth additional domain, based on appropriateness of the data.

Second, to minimise country differences in interpretations of same data, all emerging codes were classified in one Excel file code book and discussed by all researchers during face-to-face meetings, conference calls, and electronic mail correspondence. The research group agreed on the English translation of the developed codes to ensure codebook fidelity. Data collection proceeded until achievement of conceptual saturation on country level, which we defined as a state in which no new themes or codes could be generated [26]. Analyses were conducted by each country research team with the described internationally agreed format, which made it possible to perform meaningful analysis with large numbers of interviews.

Third, to maximise discussions of interpretations, exchange of views and reach of agreements, the affinity diagram method [27] was applied as an instrument in face-to-face meetings to achieve final international consensus in the research group about grouping codes and defining themes. Whereas Realist Evaluation and TICD were used as perspectives for interpretation of data, diagram affinity method was applied as an instrument to achieve consensus in analysis, as recommended in multinational qualitative research [27].

Fourth, resulting themes from the affinity diagram method were linked to the existing TICD framework domains. The general analyses were based on the themes from the third phase that had emerged nationally and internationally. To reach in-depth analyses level, the TICD concepts were not only described as domains separately, but as a second-order analysis we also explored the relations between the TICD concepts in order to catch the complexity of multinational implementation [28]. The Dutch researchers coordinated the analyses, which were subsequently evaluated and discussed by the partner researchers.

## Results

### Study population

Of the 138 professionals invited, 68 participated including 40 GPs and 28 nurses (mean response rate 49 %). The main reasons for not participating were lack of time and unanswered calls of the research team. Participant study and demographic characteristics were shown in Table 1. Participating professionals were mainly female with a mean age of 47. Catalonia needed the highest number of interviews to achieve data saturation and Poland had the lowest number of interviews, primarily because no nurses participated in the trial. Participants roughly evenly represented the three purposive sampling domains of occupation, screening performances and implementation strategy.

**Table 1:** Participating professional profiles

	Catalonia	Sweden	Poland	Netherlands	Total
N GPs	12	5	12	11	40
N nurses	10	10	0	8	28
N high performance	13	9	6	10	38
N low performance	9	6	6	9	30
N T&S	11	5	6	9	31
N no T&S	11	10	6	10	37
N FR	13	5	7	10	35
N no FR	9	10	5	9	33
N e-BI	9	6	3	11	29
N no e-BI	13	9	9	8	39
Male (%)	27	13	16	37	26
Mean age	47	52	47	44	47
Total	22	15	12	19	68

### Barriers and facilitators to implementation

Table 2 links already existing theoretical TICD concepts with practice-led affinity diagram themes that rose from the data analyses. In more detail, there are seven TICD domains [18] that included 39 relevant concepts in light of our findings, being reflected in the two left-hand columns of the table. The two right-hand columns include 57 affinity diagram themes that derived from the grouped coded data. Thereby, this table links theory and practice and consequently gives insight into important determinants for practice within this population of health professionals. An eighth additional concept was added that did not fit within the original TICD framework and was related to ‘Implementation strategy practicalities’.

**Table 2:** TICD domains and concepts linked to Affinity Diagram themes and codes

TICD Domain	Theory-led TICD concepts	• Empirically-led Affinity Diagram themes	Codes
1. Guideline factors	Cultural appropriateness	• Cultural appropriateness	SBI is not a task for PHCU_referall to specialised care outside the PHCU; no guideline available_SBI too late
	Strength of recommendation	• Barriers to adhere to the guideline	Too strict_nr of drinks; SBI does not fit in short time consult; doubts about effectiveness pro-active screening
	Compatibility	• Adherence TO guideline • Routine • Follow-up of SBI	Return to the habitual system; routine_Application of the screening in all cases; already a routine; routine_preventive activities; SBI part of the nurse’s protocol; SBI part of GP’s protocol/routine; follow-up after SBI suboptimal; policies_screening during initial general interview with every new patient; focus on alcohol addicted patients/co-addicts; focus on chronically ill patients; routine_follow-up of patients; repeat SBI
	Observability	• Facilitators to adhere to the guideline	Partly adherence to guideline; adherence to guideline; clear cut-off screening tool stimulates brief intervention; use evidence based knowledge/material; use evidence based knowledge/material – mi; adherence implementation takes a while; adherence_Initial difficulties; adherence_Simple adaptation process; interventions were feasible; feasibility_ of the instrument
	Feasibility	• Adherence to guideline • Facilitators to adhere to the guideline • Implementation of guidelines • Feasible guidelines	Example of interventions
2. Individual factors	Agreement with recommendation	• Evaluating own performance • Implementing new practice • Role perception • Screening opportunities • Barriers	Screen to make patients aware of daily drinking habit; role perception_patient motivated when given BI from a GP; performance perception_effects of SBI; performance perception_no effects of SBI; my role to start the process; role perception SBI; barrier screening_perceived_not relevant in context; role perception_to recognise signs given by a patient; it’s not my role; agreement recommendation; awareness _alcohol is not a medical problem
	Expected outcomes	• Personal motivation to participate from societal perspective • Collaboration from individual perspective • Evaluating own performance • Role perception • Professional’s expectations • I don’t care • Barriers	ODHIN outcome expectation_to catch more case positives; role perception_patients like GPs to ask about lifestyle; expectation_patient’s reaction; expectation_conformed to expectations; professional age; motivation to participate ODHIN_curiosity about the outcomes; expected MI intervention outcome_high; expected intervention outcome_low; expectation_With no initial expectations; lack of motivation to change; barriers referral_big step; GP afraid of patient’s reaction
	Emotions	• Implementing new practice • Barriers	E-health_using e-health is a personal weakness; new patient; hard to screen GP’s own friends or acquaintances
	Frustration	• Implementing new practice	ODHIN impact_more frustration
	Intention and motivation	• Personal motivation to participate from societal perspective • Training • Collaboration from individual perspective • E-health • Personal motivation individual perspective • I don’t care • Barriers	Motivation to participate in ODHIN_to help patients; ODHIN training_positive but not fully attended; Motivation to participate ODHIN_motivation for intervention; motivation to participate ODHIN_the size of alcohol problem; motivation to participate ODHIN_easier with a network; e-health_positive in e-health; e-health_barrier referral; e-health_no time to become familiar with e-health intervention; e-health_not familiair with website content; e-health_negative attitude; motivation to participate ODHIN_consider load and benefit; not motivated by financial incentives; motivation to participate ODHIN_to act pro-socially; motivation to participate ODHIN_personal interest/benefit; motivated by ODHIN financial incentives; motivation to participate ODHIN_negative; motivation to participate ODHIN_Interesting subject; not motivated to improve SBI; low patient awareness_inhibits professional; low motivation to change_inhibits professional; motivation to change_motivates BI; patient reactions_denial inhibits brief intervention;
	Learning style	• Training • Implementing new practice • Routines	ODHIN training_increases awareness of the problem; ODHIN training_temporary stimulation; ODHIN training_positive; ODHIN presence cause reminders/awareness_temporary; continuous triggers necessary for SBI; routine and practice
	Self-efficacy	• Self-efficacy	Self-efficacy in BI_high; high screening self-efficacy; self-efficacy; self-efficacy_frustration; self-efficacy in BI_moderate; performance perception_GP can always do something
	Awareness and familiarity with the recommendation	• Personal motivation to participate from societal perspective	ODHIN motivates to screen pro-active; awareness of alcohol problems; importance of screening
	Knowledge	• Training • Implementing new practice • I don’t care • Barriers • Screening opportunities	Skills thank to previous training; ODHIN impact_encouragement to introduce more prevention; previous training_don’t remember; barrier screening_language barrier; barrier screening_information from system not available; barrier BI_skills; Skills_plurimedication; Patient nightlife related with drugs/alcohol; patient known to drink too much; screen because of patient signals; skills_professional knows well patient’s medical history; importance_associated pathology; screened patients suspected of drinking alcohol; patient drunk during the visit; problem reported by family member
	Knowledge about own practice	• Collaboration from individual perspective • I don’t care • Barriers	Barrier screening_already SBI by colleague; barrier screening_other important health and other topics; barrier screening_sociodemographics; patient religious issues
	Skills needed to adhere	• Implementing new practice • Personal motivation individual perspective • Professional patient approach • Professional’s expectations • Barriers • Screening opportunities	ODHIN impact_new skills/procedures; motivation to participate ODHIN_need for more knowledge and skills; expectation_increase knowledge/skills about interventions; skills_no judgemental attitude/tolerance; skills_professional keeps motivating the patient; skills_individual approach to patient; alcohol is a sensitive issue/difficult subject; need for more knowledge & skills for SBI; performance perception_screening justified by the research project
	Capacity to plan change	• Personal motivation to participate from societal perspective • Implementing new practice	Barrier screening_economic crisis situation; ODHIN impact_introduction of new data into patients’ records
	Nature of the behaviour	• Implementing new practice	ODHIN impact_effort to perform
	Self monitoring or feedback	• Personal motivation to participate from societal perspective • evaluating own performance • implementing new practice • screening opportunities • I don’t care • Barriers	ODHIN outcome _catching patients in early stage of disease and follow-up; motivation to participate ODHIN_awareness of trivialising; satisfaction with own performance; lack of satisfaction with own performance; self-monitoring of screening; self monitoring of BI; insight SBI potential afterwards; ODHIN impact_more patient/new groups of patients screened; ODHIN presence cause reminders/awareness_own consumption behaviour; ODHIN presence cause reminders/awareness; ODHIN did not make any difference; ODHIN presence did not cause reflection on own consumption behavior; barrier screening_simply forgotten; has routine; barrier screening_experienced workload; Patient age; patient gender; physical GP’s tiredess; Screened every patient (or tried to screen)
3. Patient factors	Patient behaviour	• Patient reactions	Patient reactions; feel suspected of being a drinker; afraid/suspicies; stressed/tense; not honest; honest; frustration; defensive; surprise; relief; no objection/acceptance; negotion/trivialisation
	Patient beliefs and knowledge	• perceived patient awareness • lack of interest in E-BI	Awareness_personal decision of the patients; awareness_self-control of drinking; patient reactions_awareness guidelines; BI_difficult when patients not aware; patient reactions_don’t treat beer as alcohol; self-efficacy in BI_low/doubts if patiens will change anything; patient reactions_lack of interest e-health; patients not interested in e-BI
	Patient motivation	• Patient trust required • Motivation to change	SBI requires patient’s trust; motivation to change_Serious alcohol problem; motivation to change_Social support
	Patient preferences	• Patient reactions	Patient reactions_positive
4. Professional interactions	Communication and influence	• Decision to participate • General assessment of PHCU routines and engagement	Decision to participate in ODHIN_agreement; decision to participate in ODHIN_GP decided to participate; decision to participate in ODHIN_nurses agreed; decision to participate in ODHIN_practice nurses not involved; motivation to participate ODHIN_order or influence of other professional/supervisor/colleague, etc.; GP takes the lead in ODHIN SBI; engaged other staff in alcohol discussions than those involved in the Odhin project; team (not) on the same line; different routines among the staff
	Referral processes	• Barriers • Task division in the team • Referral	Addiction care disappointing; GP internal referral to specialised professional; nurse referral to other(s); ODHIN initiates referral option specialised nurse; GP referral to addiction care; need for low barrier referral possibilities; conditions in the PHCU_additional support
	Team processes	• Barriers • Organisation of SBI care • Task division in the team • Learning from each other • Making agreement within the practice	Recent screening; colleagues less practice/experience; organise care multidisciplinary; counseling done by other profession; care requires a specialized practice nurse; team process SBI_SBI only partly by nurse; unknown patient; practice nurses_have more time_for MI; other professionals have more time’; practice SBI in team; share experiences; lack of communication; sufficient communication; nurse not informed about procedures; agree on team objectives; agree on SBI strategy
	Undefined	• Difference in opinions	ODHIN_waisted money
5. Incentives and resources	Availability of necessary resources	• Physical working conditions in the PHCU • Difference in opinions • Tools as facilitators • Screening tool usefulness • Trigger for screening • Importance of time	Conditions in the PHCU_privacy; conditions in the PHCU_disturbances; ODHIN did not lack resources; little bureaucracy; ODHIN provides tool for BI; need for patient information_low barrier patient information; more resources in the treatment of the patient; screening instrument not within reach; advice_use available training and tools; screening tool helps to structure; advice_use screening tool; ODHIN provides screening tool; screening instrument_Suitable instrument; screening tool did not help; screening instrument_too complicated for patients; screening instrument_easy to use; screening instrument_anonymous; visible screening instrument does not stimulate; visible screening instrument stimulates; need for summary card on desk; advice_time is necessary resource; GPs want more time per patient; increase available time for extra practice nurses; time pressure inhibits BI; time pressure inhibits GP’s MI; time pressure inhibits screening; time is no barrier to screen; time is no barrier for advice; addicted patients need more time; time for creating right atmosphere; time pressure forces need for follow-up appointment
	Continuing education system	• Importance of training	Advice_continuous training; training should be organised in PHCU; more role playing; Providing training tools suitable for professionals
	Financial incentives and disincentives	• Importance of finances	No financial resources from health Insurance; finances required for practice nurse; financial incentives rewards your effort; financial incentives would create more priorities; more funds needed
	Information system	• Role in information system	Usual registration in information system; information system obligatory field; no use of information system; register SBI in information system; information system not adapted to SBI; information system not obligatory field
6. Capacity for organisational change	Assistance for organisational changes	• PHCU SBI policy • Nurses protocol for SBI	Advice_invite a consulent; practice nurse not skilled
	Monitoring and feedback	• PHCU SBI policy	Need for ongoing evaluations
	Priority of necessary changes	• PHCU SBI POLICY	Advice_SBI prioritarisation
	Regulations, rules, policies	• Systematisation of SBI • PHCU SBI policy • Nurses protocol for SBI	Policies_need for a systematic approach to disease prevention; make it part of protocol; make it part of performance indicators; Nurses protocol adapted in line with ODHIN
7. Social, political, legal factors	Economic constraints on the healthcare budget	• Increase public awareness	Advice for improving public health_society should be richer
	Influential people	• Importance of regional policy • Increase public awareness • Awareness of prevention task of primary care	The board plays an important role; advice_increase public awareness (media); advice_increase public awareness (media)_broad lifestyle; advice_increase public awareness (media)_involve environment; advice_increase school and parent awareness; little effect public campaigns; synergy effect of advice from multiple people; less ads; change social attitudes; advice_increase primary care awareness outside PHCU; increase awareness in professionals; prevention task of PHCU
	Legislation	• Need for effective policy actions • More strict legislation	Mandatory trainings for GPs’; advice_increase alcohol taxes_not effective; advice_increase alcohol taxes; advice_legislate higher age buying alcohol; advice_make alcohol less available; fear of bureaucracy
	Payer or funder policies	• Increase public awareness	Advice for improving public health_don’t waist public money on projects like ODHIN
	Undefined	• Increase public awareness • need for effective policy actions • awareness of prevention task of primary care	Advice for improving public health_use disulfiram implants; advice for improving public health_state alcohol policy is schizophrenic; raise awareness of screening, BI and available tools; build trust between GPs and patients; advice_organise peer buddy’s; increase knowledge in primary care professionals; Approach general/integral; policies_screening during initial general interview with every new patient; introduce more programs like ODHIN
8. Implementation strategy practicalities	Training and support		Caused awareness; MI requires long term practice; MI useful for other lifestyle issues; positive; preference for more factual knowledge; role playing_not favorable; temporary stimulus
	Financial reimbursement		No effect; extra motivation
	E-health		low outcome expectation; low patient motivation inhibits professional; easily accessible intervention; increases awareness; negative attitude; no time to become familiar with e-health; not applicable for elderly; not applicable for low SES; no effect

As presented in Table 2, most affinity diagram extracted themes fit the ‘individual factors’ TICD domain. Also, the TICD domains ‘professional interactions’ and ‘incentives and resources’ were important in gaining insight into the mechanisms behind the allocations. The importance of the TICD domains ‘guideline factors’, ‘patient factors’, ‘capacity for organisational change’ and ‘social, political and legal factors’ in explaining the processes of the allocations, varied per allocation. High as well as low performer views equally covered the TICD domains, whereas GPs and nurses differed in covering TICD domains. GPs held clearer views than nurses on healthcare system barriers and facilitators, which resulted in the TICD domains ‘capacity for organisational change’ and ‘social, political and legal factors’ being mainly covered from the viewpoint of GPs.

### Why?

Many professionals, both high and low screening performers and both nurses and GPs, had a positive role perception with regard to conducting SBI. Most professionals participated because of their awareness of the prevalence of alcohol-related problems and the willingness to contribute to the prevention of risky drinking. For most professionals also the likelihood of being allocated to T&S was an important motive for participation.

*Alcohol problems are really big in this area. I’ve been observing them for years.(GP, FR, low performance, PL*)

Polish and Catalan GPs reported the additional value of FR besides their willingness to contribute to the prevention of risky drinking. Dutch and Swedish GPs as well as some Catalan nurses reported not being motivated to participate for a financial reimbursement, whereas Polish and Catalan GPs felt positive about providing good care and getting paid for it as well.

There were no professionals who mentioned any e-BI related motivation to participate in the trial. Most professionals, GPs as well as nurses, were ambivalent in their attitude towards e-health. The professionals who were positive about the e-BI concept primarily thought it was useful in information provision for patients.

### How and for whom?

Aspects in three TICD domains appeared to be relevant in answering the question *how* and *for whom* T&S worked: *guideline factors*, *individual factors* and factors related to *incentives and resources*. Facilitating T&S ingredients for high SBI performance can be summarised into knowledge gained, application of tools, support offered by the trainer, and team-based education. Professionals who received training and support indicated factors that would make training and support even more effective, i.e. continuous training provision, more time to learn intervention techniques and more tailoring to experienced barriers, such as a perceived lack of time for conducting SBI. In Catalonia, Sweden and the Netherlands, training and support further raised awareness of the guidelines and stimulated many of the professionals to keep using them. Primarily for high performing GPs, training and support provided assistance in SBI application in daily practice. Most of the training and support allocated professionals perceived the guidelines to be feasible and compatible with daily practice. Most professionals in the ODHIN study wanted to know and to become skilled in *how* to implement and conduct SBI rather than be convinced of the *importance* of implementing:

*“During my education there was some attention paid to motivational interviewing, but this training was very welcome as it cleared things up, such as fine-tuning my patient approach to their phase of behaviour change according to the behaviour change matrix.” (Nurse, T&S, high performance, NL)*

High performing professionals reported that they gave more priority to SBI in their routines than before ODHIN. After attending training and support sessions, professionals felt that it was not only a matter of having time, it was also a matter of prioritising. They found that it was actually possible to frequently ask patients about alcohol consumption, even during high workload:

*“The more often you ask the questions, it will become more of a routine, it takes time to incorporate new procedures and ask the questions, but most of the time you can ask these questions during each visit” (Nurse, T&S, high performance, SWE)*

*“You have to decide beforehand whether you want to reserve time for this. Do we think it’s important enough to spend time on?” (GP, T&S + FR + e-BI, high performance, NL)*

Furthermore, learning how to raise the ‘alcohol topic’ in patient groups with varying motivation to change was appreciated in the training and support sessions. Some high performing professionals used study participation to start the conversation and to make the topic more easily accessible:

*“I stated: “We are taking part in a project aimed at people’s wellbeing”” (GP, T&S + FR, high performance, PL)*

The high performing professionals who attended training and support, reported being stimulated in discussing SBI experiences within their team. This facilitated a team approach in doing SBI:

*“We could have talked about this without the ODHIN project. But it gave us a reason to sit down and do so.” (GP, T&S, high performance, SWE)*

Furthermore, many professionals already knew about the existence of SBI tools. Even so, they were additionally informed during T&S where to find the right tools and how to apply them appropriately.

However, both low and high performing professionals reported that training and support were felt to be a temporary stimulus, and that alcohol is just one of the many important themes to discuss. Embedding SBI in the long term requires a continuous trigger, such as booster sessions. This also facilitates prioritising:

“*The emphasis on your work is on what you are currently busy with. It would be the same if I had participated in a study about cardiovascular diseases.*” *(GP, T&S + FR + e-BI, high performance, NL)*

TICD domains *individual factors*, factors related to *incentives and resources* and *social, political and legal factors* were of relevance in evaluating *how* and *for whom* a financial reimbursement strategy would work. Financial reimbursement seemed to differ in impact between Poland and Catalonia compared to Sweden and the Netherlands, mainly due to low personnel levels and salary levels.

“*Because with the cutbacks there are fewer of us and we have to…stand in for people and that’s hard, isn’t it?*” *(GP, T&S + FR, high performance, CAT)*

*“Getting an incentive is always good. If this is financial or economic, I think it could be good, but I am not completely sure about it. When you get invited to participate in a study they ask you “Do you want to participate?” and you take part voluntarily. In the end, it turns out that someone publishes an article and your name is there, that’s okay. Of course both the financial and professional incentives are important, but with the financial one you feel they treated you well.” (Nurse, T&S + FR, high performance, CAT)*

Views of Swedish and Dutch professionals allocated to financial reimbursement did not differ between high and low performers and those not being allocated to financial reimbursement. Swedish and Dutch professionals thought it was important to get paid for the care provided, but they perceived it as inferior to being a good care provider:

*“Now it is the diagnosis that brings in money, nothing out of this really benefits the patients, but that’s something for financially educated managers to calculate and put in charts and to perform some kind of statistics. What is important in healthcare is the patient.” (GP, T&S, low performance, SWE)*

Furthermore, in the ODHIN study the financial reimbursement scheme differed per country. In Poland and Catalonia, professionals were reimbursed directly, whereas in Sweden and the Netherlands reimbursement was applied on PHCU level. In Sweden and the Netherlands, professionals reported that financial resources in principle were of high importance. However, both high and low performers from these countries preferred being structurally paid for their preventive services by health insurance, rather than a temporary project based payment. They considered increased resources from health insurances required for long-term improvement of SBI:

*“I have to pay my practice nurse. If I can only pay her for other tasks [other than asking for alcohol consumption], I have to pay for it myself when she is going to ask about alcohol consumption.” (GP, T&S + e-BI, low performance, NL)*

It turned out that for all four countries patients’ lack of interest inhibited both nurses and GPs from being active in referring patients to e-BI. It neither facilitated nor guided them in providing brief interventions, as patient reactions were frequently not very promising. Therefore, face-to-face interventions were the preferred method in such cases. Consequently, the high performers did not give any e-BI related explanations for their performance levels, whereas the low performers explained the non-facilitating role of e-BI.

*“Well, I gave them the e-BI tool and asked them to access it. However, it is up to them, you can ask them to do it, but they don’t always do so. It happens very often, your role as a professional is to say ‘look, if you want more information here it is’ but in my opinion this is a challenging thing.” (Nurse, T&S + FR + e-BI, high performance, CAT)*

*“If they didn’t have a computer at home, or if they did not feel comfortable using one – then it was really not any use to recommend it to them. It was meant for those who felt that they wanted it… I don’t know if they visited the website or not.. I have no idea…” (Nurse, e-BI, low performance, SWE)*

### Under what circumstances?

The fact that many health professionals throughout the four countries participated in a trial concerning preventive services for risky alcohol consumption, raised their awareness and frequency of providing these services. That means that just putting this theme as item on the agenda already makes the professional more active in SBI, irrespective of their allocation. This was illustrated by a professional who had not received any of the implementation strategies but was still a high performer.

*“I know that before ODHIN I did not pay as much attention to this as after ODHIN. I did not have specific barriers for asking about alcohol consumption, but if you participate in this kind of project it will become more part of your automatism in anamnesis.” (GP, no strategy, high performance, NL)*

Consequently, before being able to receive a state of SBI routine, one should be increasingly aware of their SBI activities. Referral opportunities could provide stimulating thoughts for professionals to take up this activity. Another important precondition to make it part of a routine, is to include it in protocols and to set reminders.

*“Include it in your protocol. Every time you see it [on your screen], you will be reminded.” (Nurse, T&S + FR + e-BI, high performance, NL)*

However, there are some preconditions that can facilitate or hinder successful a implementation of brief interventions, such as information systems. As countries differed in their information systems, the role of the information system as a facilitator varied.

*“Yes… it has facilitated our work a lot because we already had it implemented in our computerised medical record (E -CAP)… and … and this is the usual computerised tool that we always use, as a result of this it has been much easier.” (GP, T&S + FR + e-BI, High performance, CAT)*

*“I do register, but it’s a bit difficult as we do not have an appropriate ICPC [declaration] code” (GP, FR + e-BI, low performance, NL)*

Subsequently, professionals frequently reported high workloads, which caused T&S not to be sufficient to increase performance.

*There are not enough GPs … more time and more funds should be reserved … e.g. one extra hour per week for preventive visits should be founded by the National Health Fund (GP, T&S, low performance, PL)*

Another inhibiting factor was that the alcohol subject seemed to compete with other lifestyle prevention themes. For example alcohol received less media attention compared to other lifestyle prevention themes:

*“For professionals, you have to notice it more, read about it more, pay more attention to it in the media and literature. (…) The lobby for quitting smoking is much bigger than the lobby for drinking less.” (GP, FR + e-BI, low performance, NL)*

### Second-order analysis: relations between framework domains

Many drivers for the trialled SBI implementation strategies were found in the TICD domains ‘Individual health professional factors’ and ‘incentives and resources’. However, these were embedded in other TICD domains to influence SBI implementation in daily practice. In particular, political culture – part of ‘social, political and legal factors’ domain – is such an important contextual factor that exert the SBI implementation in daily practice. To create an environmental SBI culture, a facilitating political and social culture is essential:

*“The state earns most on alcohol and tobacco. So limiting consumption is against its economic interests.” (GP, T&S + FR, high performance, PL)*

*“There is a social acceptance for drinking.” (GP, T&S + FR, high performance, PL)*

Furthermore, the organisational environment challenges the SBI implementation, even when implementation strategies seem to work at the individual level i.e.:

“The system of work should be changed. Besides alcohol interventions, interventions on nicotine, obesity, physical activity should be conducted. And I have 10–15 minutes per patient.”*(GP, no strategy, low performance, PL)*

*“I do register [SBI], but it’s a bit difficult as we do not have a good ICPC code [for health insurance declaration].” (GP, T&S + FR + e-BI, high performance, NL)*

Implicitly, responses of both nurses and GPs show their perceived responsibility in SBI, yet as part of the SBI responsibility as society together. Despite their intrinsic motivation to prevent patients from alcohol-related disabilities, GPs and nurses feel more rationale for selective screening rather than opportunistic screening:

*“When there are analytical alterations or when there’s a sonogram that shows something, when there’s a pathology behind it (…), it’s easier to focus on it.” (nurse, FR, low performance, CAT)*

These insights taken cumulatively, it seems that implementation strategies should be applied in other healthcare settings as well, next to primary healthcare. The ODHIN study tested implementation strategies at micro-level and meso-level. Implementation determinants on the macro-level as described by TICD domains seemed to challenge the tested implementation strategy influences. Therefore it raises the need for an integrative SBI approach to take broader than primary healthcare.

## Discussion

The aim of this study was to explore, according to professionals’ opinions, *why, how, for whom* and *under what circumstances* the implementation strategies tested in ODHIN increased or did not increase SBI. T&S improved knowledge and skills in team-based approach and taught professionals to prioritise SBI. Continuous provision, sufficient time for learning intervention techniques and tailoring to individual experienced barriers, were important perceived facilitators. Catalan and Polish professionals perceived financial reimbursement as an additional stimulating factor, as SBI rates were smoothened by personnel levels and salary levels. Structural payment for preventive services, rather than a temporary project based payment, might have further increased the SBI rates. Implementing e-BI seem to require more guidance than was delivered in ODHIN, for example in connection with unmotivated patients. Other preconditions for SBI in routine care, irrespective of the allocation, are frequent exposure of this topic in media and guidelines; information systems that facilitate SBI (e.g. screening programmes); and having SBI in protocol-led care. However, despite having identified facilitating factors on the micro-individual level, the macro-level in which SBI is augmented to be implemented includes important barriers. These were mainly related to politics and social culture.

The purposive sampling strategy in this study was based on occupation, implementation strategy and screening performances. This qualitative study showed that allocation to T&S or FR influenced professionals’ views, whereas e-BI did not seem to make any difference. Occupation did not seem to influence views, perceptions and opinions, although GPs reported higher importance of financial resources and experienced barriers in implementing routine SBI. Furthermore, GPs had clearer views on the barriers and facilitators of the healthcare system, which we perceive a result of different tasks and functions by professionals in the organisation of primary healthcare. Tailored strategies seem important, also with regard to who makes decisions and who is financially responsible. Furthermore, despite positive SBI outcomes after T&S and FR during high workloads, time constraints remained. This indicated the need for more profound changes in the structure of the healthcare organisation to facilitate further SBI improvements in primary healthcare.

In line with the literature, our study confirms that very few professionals used e-health in patient care [29, 30]. An important barrier for implementing e-BI was that professionals from all countries were mixed in their trust in e-BI in principle and they noticed that their patient population was not interested in e-BI. Despite the effectiveness of SBI self-help via internet in principle [31], our findings imply that more efforts might be required in getting the facilitated e-BI access embedded into daily primary healthcare practice. For example, professionals seem to require clearer guidance in how the facilitated access can decrease their workload by using e-BI interventions that have proved to be effective [29, 32]. In the ODHIN programme offering e-BI might have been too much a matter of being ‘dropped’ as a strategy rather than personal guidance in using it with a population who is less familiar with the internet, such as the elderly or in a population with a low motivation to change alcohol consumption, as experienced during ODHIN.

When implementing lifestyle interventions such as alcohol related screening and brief interventions, it is important to address sustainable funding of services [33]. In the United Kingdom (UK), the Quality and Outcomes Framework (QOF) is a reimbursement scheme in which payment is based on fee-for-service and capitation systems rather than related to quality of care [34]. After 20 systematic reviews and one systematic reviews of systematic reviews [35], it is clear that pay for performance can be effective. However, policy makers should be warned that effects may be only realised on short-term and may be not as large as one may wish [35]. Pay for performance has potential, but it is not a “magic bullet”. To achieve sustainable changes, it needs to be combined with other quality improvement initiations [35].

Of the total 57 concepts included in the seven domain TICD checklist framework [23], 39 concepts were covered in this study. Non-covered concepts were mainly associated with topics not relevant in the study context, such as corruption or political stability. For Poland specifically, it is no surprise that guideline topics were hardly covered, as no official guidelines exist. Furthermore, one can imagine that healthcare professionals talk more easily about their daily practice than about topics that are more general and policy-related, such as topics with *social, political and legal factors*. These topics were more indirectly covered in the second-order analysis. Other professional disciplines such as managers and policy makers could add on the more meso- and macro-perspective. In addition, more context-related items should receive attention– e.g. Poland mainly has solo-practitioners (GPs) who are not able to refer to other providers in the practice, or differences in country-specific guidelines to adapt SBI procedures.

Only four themes identified in the analysis did not match with the TICD checklist. These four were either very specific, such as opinions regarding specific medicaments, or very generally formulated, such as with increasing public awareness. However, these were of minor importance in answering the research question.

There are caveats as well as strengths to mention. The interview questions about allocation experiences and views varied across participating countries. Sweden and the Netherlands pro-actively asked professionals about their experiences with all three implementation strategies. Catalonia covered all three but focused on T&S, whereas Poland mainly focused on the project generally and asked for further explanation when any of the strategies was raised by the professionals themselves. Despite this systematic difference, there were minor differences in FR and e-BI data saturation due to the equally represented allocations. The e-BI coverage in the results section is less compared to FR and T&S. Despite reaching data-saturation, the participating professionals did not share much e-BI related data. Consequently, this data limitation impedes to provide full answer on the research questions related to e-BI and therefore deserves further research. Another caveat is the selection of professionals who are likely to be more motivated to prevent alcohol problems, compared to the greater primary healthcare professional population. This could make the implementation strategies less powerful, and it could make the conditional circumstances described of greater importance.

A strength of the study was the use of different country contexts when striving after code homogenisation of emerging themes in light of the Realist Evaluation built international code book. The Realist Evaluation then helped to distinguish between a context and a mechanism [36]. For instance, there were differences in the state of the art regarding SBI implementation. Catalan, Swedish and Dutch professionals already paid (some) attention to lifestyle prevention themes including alcohol, while many Polish professionals did not pay any attention to alcohol SBI before participating in ODHIN, which is in line with the absence of a Polish national guideline. Other examples are differences in countries’ cutbacks in personnel and salaries, policies and social progress towards SBI implementation differed, which made comparisons sometimes difficult. To increase meaningful analysis of the data on international level, we organised face-to-face discussions and conference calls to agree on scientific value of our findings over all four countries. In addition, a major strength of the study is that the approach of the realist evaluation was combined with the TICD framework analysis. The Realist Evaluation perspective was developed to unpack the ‘how’ and ‘why’ questions and illuminate the many, varied and interdependent, mechanisms by which interventions may work (or fail to work) in different contexts in education [23, 24]. This makes sense with regard to implementation programmes, as these are often complex and multifaceted [28, 37] and enabled the second-order analysis [28]. The interpretative approach of the realist evaluation [24] was considered to be appropriate in evaluating not only why our implementation strategies worked or did not work, but also in which type of context and in which situation. Another strength is that this is the first qualitative study evaluating implementation strategies with regard to SBI, next to numerous qualitative studies on this topic as presented in a review of Johnson et al. [21].

An issue that deserves consideration is the sustainability of the implementation efforts. Future implementation programmes should provide booster training sessions to update knowledge, to set alcohol SBI on the agenda, to maintain SBI skills and institutional support. Also when the professional team formation changes, booster session could be important to reformulate different professional roles within teams. Second, structural payment for preventive services, rather than a temporary project based payment, is important for both short term and for long term. More importantly, implementation strategies on the macro level should be applied to influence the societal and political culture. Only then, initiatives on the micro and meso-level can be highly successful. Successful e-BI strategies deserve further research attention, as the limited e-BI related data in this study impedes to provide full answer on the research questions related to e-BI.

We believe that the present study considerably advanced our understanding of alcohol SBI implementation processes in different contexts. A review of Chaudoir et al. [38] indicated that organisation, professional and innovation-level constructs have the most usable measures for implementing health innovations, whereas structural and patient-level constructs have the least usable measures [38]. Implementing guidelines like alcohol SBI, can be regarded as a ‘health innovation’. When we compare the review results of Chaudoir et al. with the results from the present study, we found that most findings were in agreement with the indicated measures. Factors related to guidelines, individual professionals, incentives and resources as well as a capacity for organisational change were most important in reaching the aim of this study. This study adds the importance of meso- and macro-influences when implementing potentially powerful SBI drivers.

## Conclusions

To summarise, T&S essential implementation ingredients seemed to be gained knowledge and skills, team-based training and learning to prioritise SBI during high workloads. FR directed SBI motivations appeared to be highly determined by country context and were influenced by the way reimbursement was provided and by the reimbursing parties. Structural payment is an important precondition. Despite e-BI proved effectiveness in previous lifestyle studies [31], this study showed that professionals require clear guidance in how the facilitated access can improve SBI in routine practice. To give a complete answer on the e-BI research question of this manuscript, additional research is needed.

These insights gained help to further tailor T&S, FR, and e-BI implementation strategies in order to achieve maximum gains in increasing alcohol SBI and risky alcohol consumption. However, the macro-level in which SBI is augmented to be implemented has an influential role. High potential implementation strategies on the micro level could get nullified due to influences from the macro- level such as societal and political culture. Focusing only on the primary healthcare setting seems insufficient and a more integrated SBI culture, together with meso- and macro-focused implementation process is requested.

## Abbreviations

COREQ, consolidated criteria for reporting qualitative research; e-BI, internet-based method of delivering advice; FR, financial reimbursement; GP, general practitioner; PHCU, primary healthcare unit; SBI, screening and brief intervention; T&S, training & support; TICD, tailored implementation for chronic diseases
